# Pneumonia is an independent risk factor for pyogenic liver abscess: A population-based, nested, case-control study

**DOI:** 10.1371/journal.pone.0178571

**Published:** 2017-06-01

**Authors:** Sai-Wai Ho, Chao-Bin Yeh, Shun-Fa Yang, Han-Wei Yeh, Jing-Yang Huang, Ying-Hock Teng

**Affiliations:** 1 Department of Emergency Medicine, School of Medicine, Chung Shan Medical University, Taichung, Taiwan; 2 Department of Emergency Medicine, Chung Shan Medical University Hospital, Taichung, Taiwan; 3 Institute of Medicine, Chung Shan Medical University, Taichung, Taiwan; 4 Department of Medical Research, Chung Shan Medical University Hospital, Taichung, Taiwan; 5 School of Medicine, Chang Gung University, Taoyuan City, Taiwan; Beijing Cancer Hospital, CHINA

## Abstract

**Background:**

Bacteremic pneumonia is considered a potential cause of distal organ abscess formation. Therefore, we hypothesize that pneumonia is a risk factor for pyogenic liver abscess (PLA).The aim of this study is to explore the association between pneumonia and PLA.

**Methodology/Principal findings:**

A nationwide, population-based, nested, case–control study was conducted using data from the Taiwan National Health Insurance Research Database. In total, 494 patients with PLA and 1,976 propensity score matched controls were enrolled. Conditional logistic regression was used to estimate adjusted odds ratios (aORs) in patients with exposure to pneumonia before PLA. After matched and adjusted for confounding factors including age, sex, urbanization, income, chronic liver disease, alcohol-related disease, biliary stone, chronic kidney disease, diabetes mellitus, chronic liver disease, and cancer, hospitalization for pneumonia remained an independent risk factor for PLA with an aORs of 2.104 [95% confidence interval (CI) = 1.309–3.379, *p* = 0.0021]. Moreover, the aORs were significantly higher among patients hospitalized for pneumonia within 30 days (aORs = 10.73, 95% CI = 3.381–34.054), 30–90 days (aORs = 4.698, 95% CI = 1.541–14.327) and 90–180 (aORs = 4.000, 95% CI = 1.158–13.817) days before PLA diagnosis.

**Conclusion:**

Pneumonia is an independent risk factor for subsequent PLA. Moreover, hospitalization for pneumonia within 180 days before PLA diagnosis was associated with an increased risk of PLA.

## Introduction

Pyogenic liver abscess (PLA) is a potentially fatal bacterial infection of the hepatic parenchyma, with a 5.6%–23% mortality rate [1, 2]. Moreover, complications, such as metastatic infection to the lungs, central nervous system, and eyes, increase patient morbidity and mortality [3, 4]. Most PLA cases develop because of systemic bacteremia or intraabdominal infections [5]. An immunocompromised status, diabetes mellitus, liver cirrhosis, and advanced age are well-known predisposing risk factors for PLA [6, 7].

However, the etiologies of and risk factors for PLA have continued to evolve; recently reported risk factors include zolpidem and proton pump inhibitor use and splenectomy [8–10]. In Taiwan, the most common pathogen of PLA has also changed from *Escherichia coli* to *Klebsiella pneumoniae* since the 1980s [11]. Notably, in addition to causing PLA, *K*. *pneumoniae* is also a dominant pathogen of bacteremic community-acquired pneumonia in Taiwan [12]. In South Africa, pneumonia accounted for up to 62% of *K*. *pneumoniae* bacteremia [13]. Furthermore, many pneumonia survivors may acquire bacterial infections after their original pneumonia infection. Brain abscess, psoas muscle abscess, para-aortic arch abscess, thyroid abscess, splenic abscess, and infraorbital abscess have been reported after pneumonia [14–19]. Bacteremic pneumonia is considered a potential cause of distal organ abscess formation. Therefore, we hypothesize that pneumonia is a risk factor for PLA.

Understanding the risk factors for PLA is clinically crucial because rapid diagnosis followed by appropriate management of PLA can improve patient outcomes and prevent complications. Here, we conducted a case–control study by using a national database from Taiwan and explored the association between pneumonia and PLA.

## Materials and methods

### Database and settings

This nested case–control study was conducted using registration and claims data of 2009–2013 obtained from the Longitudinal Health Insurance Database 2010 (LHID2010), a subset of the National Health Insurance (NHI) Research Database (NHIRD), managed by the Taiwanese National Health Research Institutes. This dataset contains all information regarding sociodemographic status such as sex and date of birth; outpatient, inpatient, and emergency care; and prescription drugs of 1 million of the NHIRD beneficiaries, randomly sampled from the 2010 registry of 23 million of the NHIRD beneficiaries. The disease diagnosis is based on the International Classification of Diseases, Ninth Revision, Clinical Modification (ICD-9-CM) codes. The diagnosis coding in this dataset is highly reliable because all insurance claims have been monitored by medical reimbursement specialists and through peer review. Our study protocol was approved by the Institutional Review Board of Chung Shan Medical University Hospital (CSMU No.: 15061). The requirement for written informed consent from the participants was waived because the LHID2010 contains deidentified data.

### Study population

Adult patients aged >20 years diagnosed as having new onset PLA (ICD-9-CM: 572.0) between January 2010 and December 2013 were categorized as the case group. The date of first hospitalization for PLA was defined as the index date. Patients with previous PLA diagnoses in the data set before January 2010 were excluded (n = 141).

**Fig 1:**
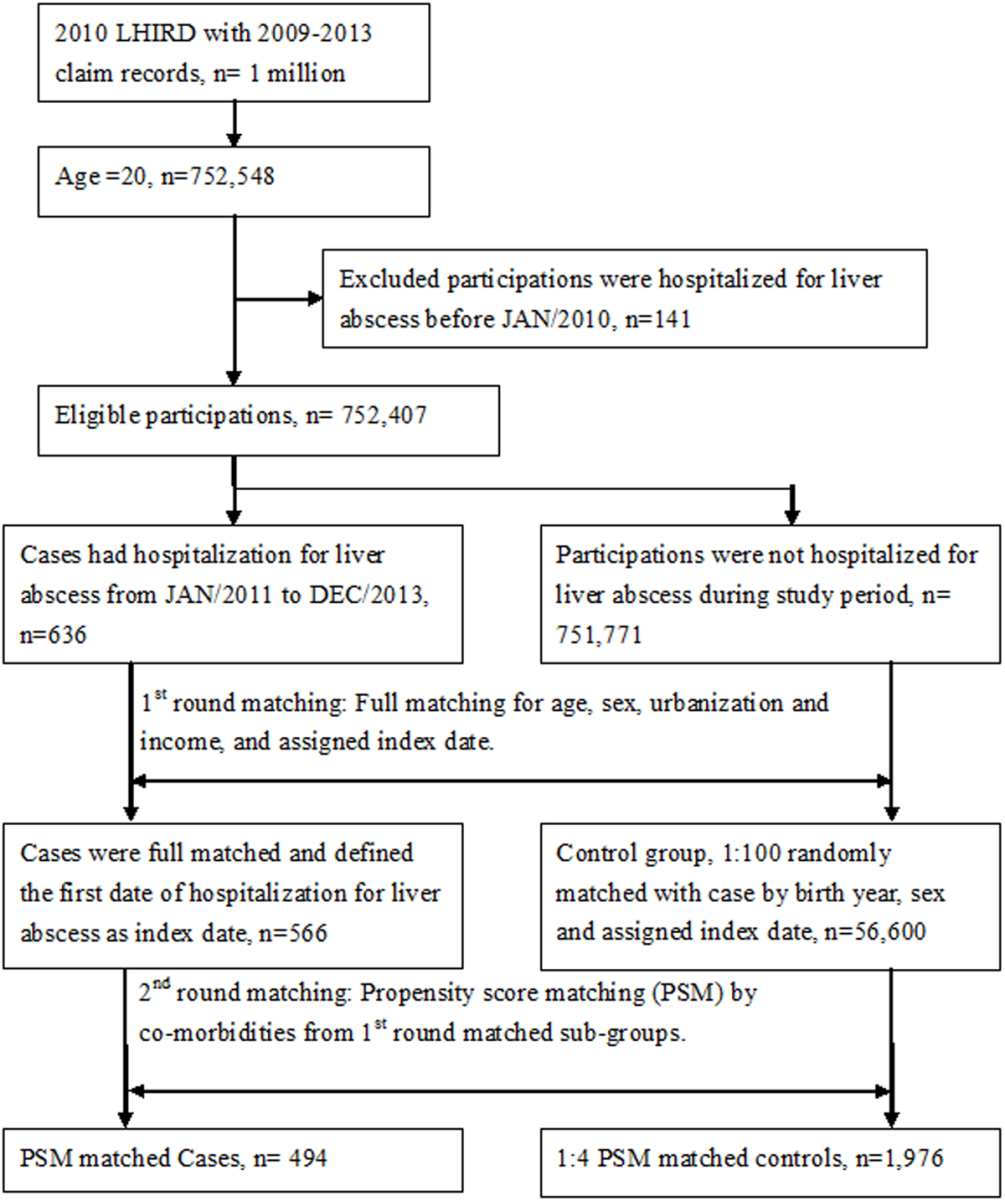
Flow chart for pyogenic liver abscess (PLA) patient selection.

For each PLA case, the first round matching was conducted to select controls at a 1:100 full matching by age, sex, urbanization and income. The second round matching was applied to reduce the confounding of co-morbidities such as diabetes mellitus (DM; ICD-9-CM: 250), chronic liver disease (ICD-9-CM: 456.0–456.2, 571.2, and 571.4–571.6), chronic kidney disease (CKD; ICD-9-CM: 582, 583, 585, 586, and 588), biliary stone (ICD-9-CM: 574), chronic obstructive pulmonary disease (COPD; ICD-9-CM: 490, 491, 492, and 494–496), alcohol-related disease (ICD-9-CM: 291, 303, 305.0, 357.5, 425.5, 535.3, 571.0–571.3, 655.4, and 760.71), and cancer (ICD-9-CM: 140–208) by 1:4 propensity score matching in each first round matching sub-groups. Patients with viral pneumonia (ICD-9-CM: 480, 487, 488 and 488.1) were excluded. Fig 1 illustrates our study framework.

### Definition of pneumonia

We defined pneumonia (ICD-9-CM: 481, 482, 483, and 485–486) as the primary diagnosis, which required hospital admission and intravenous antibiotic treatment. A study demonstrated that the identification of inpatient pneumonia using this method has a positive predictive value of 88% [20].

### Statistical analysis

Categorical variables were delineated as numbers and percentages and compared using the chi-squared or Fisher exact test, where appropriate. Continuous data were delineated as means ± standard deviations and compared using the independent *t* test. Conditional logistic regression was used to estimate crude and adjusted odds ratios (aORs) with a 95% confidence interval (CI) for case group compared with control group. In multivariate analysis, we adjusted for comorbidities that may have predisposed a patient to PLA. In the subsequent analyses, we stratified pneumonia events into the number of patients hospitalized for pneumonia and duration of the hospitalization. Statistical analysis was performed using SPSS (version 18.0; SPSS Inc., Chicago, IL, USA) and SAS (version 9.4; SAS Institute, Cary, NC, USA). A *p* value <0.05 indicated statistical significance.

## Results

**Fig 2:**
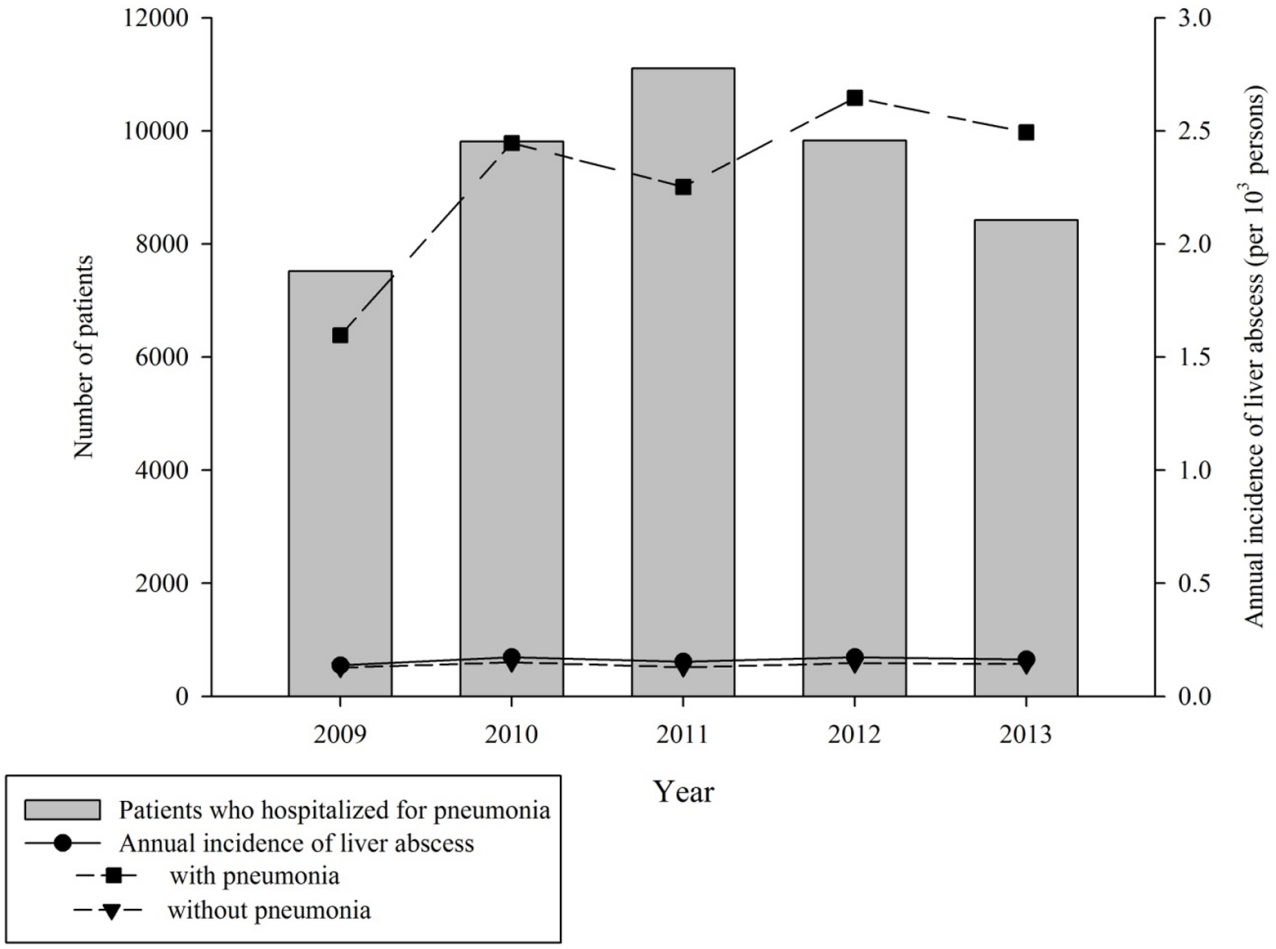
Annual incidence of pyogenic liver abscess (PLA) in 2009–2013.

After propensity score matching, a total of 494 cases of new onset PLA and 1,976 control patients without PLA were enrolled in this study for final analysis. Table 1 lists the demographic characteristics of the patients and their distribution in the case and control groups. The mean age was 60.11 ± 14.43 and 60.09 ± 14.46 years in the case and control group, respectively; no significant difference was noted in age, sex, and socioeconomic status between the two groups after matching. However, compared with the control group, the case group had a significantly higher proportion of the biliary stone (4.05% vs 2.02%) and previous hospitalization for pneumonia (5.67% vs 2.83%). During the study period, the trend of incidence of PLA was correlated to the trend of incidence of hospitalization for pneumonia (Fig 2).

**Table 1:** Characteristics of case and control groups.

	1^st^round matching			2^nd^ round matching, PSM		
	Control	PLA cases		Control	PLA cases	
	n = 56,600	n = 566	p value^+^	n = 1,976	n = 494	p value^#^
Age in index date (Mean± SD)	60.4±14.43	60.4±14.41	0.9960	60.11±14.43	60.09±14.46	0.9833
Sex			1.0000			1.0000
Female	21,400(37.81%)	214(37.81%)		764(38.66%)	191(38.66%)	
Male	35,200(62.19%)	352(62.19%)		1,212(61.34%)	303(61.34%)	
Urbanization			1.0000			1.0000
Urban	35,500(62.72%)	355(62.72%)		1,288(65.18%)	322(65.18%)	
Sub-urban	17,500(30.92%)	175(30.92%)		580(29.35%)	145(29.35%)	
Rural	3,600(6.36%)	36(6.36%)		108(5.47%)	27(5.47%)	
Income			1.0000			1.0000
Independent	15,000(26.50%)	150(26.50%)		496(25.1%)	124(25.1%)	
≦17280	12,500(22.08%)	125(22.08%)		452(22.87%)	113(22.87%)	
17280–21000	14,500(25.62%)	145(25.62%)		488(24.70%)	122(24.70%)	
21000–34800	6,100(10.78%)	61(10.78%)		212(10.73%)	53(10.73%)	
>34800	8,500(15.02%)	85(15.02%)		328(16.60%)	82(16.60%)	
Co-morbidities						
Hospitalized for pneumonia	1255(2.22%)	34(6.01%)	< .0001	56(2.83%)	28(5.67%)	0.0019
COPD	4381(7.74%)	59(10.42%)	0.0176	157(7.95%)	48(9.72%)	0.2018
Alcohol-Related Disease	392(0.69%)	17(3.00%)	< .0001	20(1.01%)	2(0.4%)	0.1988
Biliary stone	1013(1.79%)	57(10.07%)	< .0001	40(2.02%)	20(4.05%)	0.0090
Chronic kidney disease	2291(4.05%)	56(9.89%)	< .0001	163(8.25%)	37(7.49%)	0.5801
Diabetes	9595(16.95%)	197(34.81%)	< .0001	639(32.34%)	149(30.16%)	0.3533
Chronic liver diseases	3351(5.92%)	82(14.49%)	< .0001	182(9.21%)	49(9.92%)	0.6286
Cancer	2822(4.99%)	77(13.6%)	< .0001	160(8.10%)	41(8.30%)	0.8830

The 1^st^ round matching was conducted to select controls by age, sex, urbanization and income, and assigned index date by 1:100 full matching from all eligible participations. The 2^nd^ round matching was completed to reduce the confounding of co-morbidities by 1:4 propensity score matching (PSM) in each 1^st^ round matching sub-groups.

^+^ Test of difference between case and control in 1^st^ round matched subjects.

^#^ Test of difference between case and control in 2^nd^ round matched subjects

Table 2 showed the result by unconditional logistic regression analysis, hospitalization for pneumonia was an independent risk factor for PLA (aORs = 2.136, 95% CI = 1.289–3.537, *p* = 0.0032) after the adjustment for confounding factors, such as age, sex, urbanization, income, co-morbidities(including, COPD, alcohol-related disease, biliary stone, CKD, DM, chronic liver disease, and cancer). Another risk factors for PLA was biliary stone (aORs = 1.959, 95% CI = 1.112–3.452).

**Table 2:** Unconditional logistic regression of estimated odds ratios for PLA after propensity score matching.

	aOR	95% C.I.	p value
Age (per 1 year)	0.997	0.989–1.006	0.5493
Sex			
Female	Reference	-	-
Male	1.002	0.81–1.24	0.9847
Urbanization			
Urban	Reference	-	-
Sub-urban	0.998	0.793–1.256	0.9866
Rural	0.993	0.623–1.583	0.9756
Income			
Independent	Reference	-	-
≦17280	0.983	0.728–1.327	0.909
17280–21000	1.007	0.744–1.363	0.9644
21000–34800	0.998	0.678–1.468	0.9917
>34800	0.992	0.703–1.401	0.9646
Co-morbidities			
Hospitalized for pneumonia	2.136	1.289–3.537	0.0032
COPD	1.087	0.751–1.573	0.6591
Alcohol-Related Disease	0.354	0.081–1.543	0.1669
Biliary stone	1.959	1.112–3.452	0.0199
Chronic kidney disease	0.894	0.602–1.327	0.5779
Diabetes	0.917	0.731–1.150	0.4530
Chronic liver diseases	1.126	0.803–1.579	0.4902
Cancer	0.990	0.685–1.432	0.9576

aOR, adjusted Odds Ratio, was estimated by unconditional logistic regression while controlled by age, sex, urbanization, income, and other co-morbidities

Our main findings, the results of conditional logistic regression that adjusted for co-morbidities, are shown in Table 3. The aORs of PLA for exposure to pneumonia within 2 year prior to index date was 2.104 (95% CI = 1.309–3.379) in model 1. To further explore the association between hospitalization for pneumonia and PLA onset, the time interval between date of last hospitalization for pneumonia and the index date was calculated for each patient. The aORs for the time interval between hospitalization for pneumonia and PLA onset were listed, including hospitalized for pneumonia 30 days (aORs = 10.73, 95% CI = 3.381–34.054), 30–90 days (aORs = 4.698, 95% CI = 1.541–14.327) and 90–180 (aORs = 4.000, 95% CI = 1.158–13.817) days before the index date, were significantly higher than those of patients not hospitalized for pneumonia in model 2.

**Table 3:** Risk of pyogenic liver abscess in pneumonia patients by time interval between hospitalization for pneumonia and PLA onset.

	aOR	95% C.I.	p value
Model 1- Hospitalized for pneumonia within 2 years prior to index date			
No (n = 2,386)	Reference	-	-
Yes (n = 84)	2.104	1.309–3.379	0.0021
Model 2- Time interval			
No hospitalization for pneumonia (n = 2,386)	Reference	-	-
-1 month to index date (n = 16)	10.73	3.381–34.054	< .0001
-3 months to -1 month (n = 14)	4.698	1.541–14.327	0.0065
-6 months to -3 month(n = 10)	4.000	1.158–13.817	0.0284
-12 months to -6 months(n = 15)	1.009	0.284–3.587	0.9887
-24 months to -12 months(n = 29)	0.318	0.075–1.341	0.1187

aOR, adjusted Odds Ratio, was estimated by conditional logistic regression and adjusted for co-morbidities

Table 4 indicates the five specific models we used for subgroup analysis to explore the risk of PLA and number of pneumonia admissions within 1, 3, 6, 12, and 24 months before the index date. These five models demonstrated that patients having an increased number of hospitalizations for pneumonia before the index date had a significantly higher aORs for the development of PLA.

**Table 4:** Risk of pyogenic liver abscess in pneumonia patients by number of hospitalizations for pneumonia at specific time-point before index date.

	aOR	95% C.I.	p value
Model 1: within 1 month before index date			
No hospitalization for pneumonia (n = 2386)	reference	-	-
≧1 time (n = 16)	10.453	3.315–32.966	< .0001
Model 2: within 3 month before index date			
No hospitalization for pneumonia (n = 2440)	reference	-	-
1 time (n = 26)	6.733	2.832–16.007	< .0001
≧2 times (n = 4)	12.000	1.248–115.362	0.0314
Model 3: within 6 month before index date			
No hospitalization for pneumonia (n = 2430)			
1 time (n = 31)	5.480	2.630–11.422	< .0001
≧2 times (n = 9)	9.585	2.345–39.177	0.0017
Model 4: within 1 year before index date			
No hospitalization for pneumonia (n = 2415)			
1 time (n = 40)	3.147	1.662–5.959	0.0004
≧2 times (n = 15)	7.251	2.404–21.867	0.0004
Model 5: within 2 years before index date			
No hospitalization for pneumonia (n = 2386)			
1 time (n = 64)	1.767	1.023–3.053	0.0412
≧2 times (n = 20)	3.753	1.471–9.574	0.0056

aOR, adjusted Odds Ratio, was estimated by conditional logistic regression and adjusted for co-morbidities

Table 5 shows the underlying comorbidities of patients who were hospitalized for pneumonia in PLA and no PLA group. Compare with no PLA group, the ORs of pneumonia among all comorbidities were not higher in PLA group.

**Table 5:** Odds ratio of pneumonia and patient comorbidities in PLA and non-PLA group.

	No PLA			PLA		
	No Pneumonia	Pneumonia	OR (95% C.I.)	No Pneumonia	Pneumonia	OR (95% C.I.)
	n = 1920	n = 56		n = 466	n = 28	
Co-morbidities						
COPD	130(6.77%)	27(48.21%)	12.82(7.37–22.3)	33(7.08%)	15(53.57%)	15.14(6.65–34.47)
Alcohol-Related Disease	18(0.94%)	2(3.57%)	3.91(0.89–17.29)	2(0.43%)	0(0%)	-
Biliary stone	37(1.93%)	3(5.36%)	2.88(0.86–9.64)	19(4.08%)	1(3.57%)	0.87(0.11–6.76)
Chronic kidney disease	146(7.6%)	17(30.36%)	5.30(2.92–9.59)	32(6.87%)	5(17.86%)	2.95(1.05–8.27)
Diabetes	607(31.61%)	32(57.14%)	2.88(1.68–4.94)	137(29.4%)	12(42.86%)	1.80(0.83–3.91)
Chronic liver diseases	176(9.17%)	6(10.71%)	1.19(0.50–2.81)	47(10.09%)	2(7.14%)	0.69(0.16–2.98)
Cancer	151(7.86%)	9(16.07%)	2.24(1.08–4.67)	35(7.51%)	6(21.43%)	3.36(1.28–8.83)

COPD = chronic obstructive pulmonary disease. PLA = pyogenic liver abscess.

Table 6 lists the etiology and pathogens of pneumonia in patients hospitalized for pneumonia. Only the infection of K. pneumoniae demonstrated a significant difference between the case and control groups (*p* = 0.0383).

**Table 6:** Etiology and pathogens of pneumonia in patients hospitalized for pneumonia.

Pneumonia due to microbial infection	ICD-9 code	Control N = 68	Liver abscess N = 39	p value
Klebsiella pneumoniae	482.0, 041.3	2(3.57%)	5(17.86%)	0.0383
Pseudomonas	482.1, 041.7	5(8.93%)	1(3.57%)	0.6584
Hemophilus influenzae	482.2, 041.5	0(0.00%)	0(0.00%)	1.0000
Streptococcus	482.3, 041.0	0(0.00%)	0(0.00%)	1.0000
Staphylococcus	482.4, 041.1	0(0.00%)	2(7.14%)	0.1084
Anaerobes	482.81, 041.84	0(0.00%)	0(0.00%)	1.0000
Escherichia coli	482.82, 041.4	2(3.57%)	0(0.00%)	0.5502
Legionnaires' disease	482.84	0(0.00%)	0(0.00%)	1.0000
Pneumococcus	041.2, 481	0(0.00%)	0(0.00%)	1.0000
Proteus	041.6	0(0.00%)	0(0.00%)	1.0000
Mycoplasma	041.81, 483.0	1(1.79%)	0(0.00%)	0.4769
Bacteroides fragilis	041.82	0(0.00%)	0(0.00%)	1.0000
Clostridium perfringens	041.83	0(0.00%)	0(0.00%)	1.0000
Helicobacter pylori	041.86	0(0.00%)	0(0.00%)	1.0000
Chlamydia	483.1	1(1.79%)	0(0.00%)	1.0000
Other gram-negative bacteria	482.83, 041.85	5(8.93%)	2(7.14%)	1.0000
Other specified bacteria	482, 482.89,482.9, 483.8, 041.89, 041.8, 041.9	2(3.57%)	0(0.00%)	0.5502

## Discussion

According to our review of relevant literature, this is the first study to demonstrate that previous hospitalization for pneumonia is an independent risk factor for subsequent PLA (aORs = 2.104, *p* = 0.0021) by using a nationwide administrative database. Our findings also demonstrate that time interval between hospitalization for pneumonia and PLA onset as well as the number of hospitalizations for pneumonia is associated with the risk of PLA.

A potential explanation for our results is the bacterial seeding that occurs in the hepatic parenchyma from the bloodstream during pneumonia. In Taiwan, *Mycoplasma pneumoniae*, *Chlamydia pneumoniae*, *Streptococcus pneumoniae*, and *K*. *pneumoniae* are common pathogens of community-acquired pneumonia [21]. Among these micro bacteria, *K*. *pneumoniae* is the pathogen most commonly isolated from patient with pneumonia of higher severity [12, 21]; it is also the major cause of bacteremic pneumonia and PLA in Taiwan. The situation in Taiwan is different from Western countries: the prevalence of liver abscess is lower in most Western populations [22]. In the United States, the most common bacterial pathogens of pneumonia are *Strep*. *pneumoniae*, *M*. *pneumoniae*, *Staphylococcus aureus*, and *Legionella pneumophila* [23]. Notably, *K*. *pneumoniae* infection is relative uncommon in Western populations [13]. The high rate of infection of the K1 and K2 capsular serotype strains of *K*. *pneumoniae* in East Asian countries may explain the aforementioned association between pneumonia and PLA in Taiwan [24–26]. Our study also demonstrated a significant difference in *K*. *pneumoniae* pneumonia between the case and control groups, but these groups contained only 5 and 2 patients with *K*. *pneumoniae* infection, respectively (Table 6). This low prevalence of *K*. *pneumoniae* infection may have been underestimated because of the nature of the LHID2010. This dataset provides only the first four major ICD-9-CM codes of each patient for analysis. Here, we used ICD-9-CM codes of 482.0 and 041.3 to represent *K*. *pneumoniae* infection. Because this diagnostic code does not represent a specific disease, not all physicians key in this code but only the major disease code, potentially leading to selection bias. Furthermore, ICD-9-CM codes for infections of other bacteria, such as *M*. *pneumoniae* (ICD-9-CM: 483.0 and 041.81), *C*. *pneumoniae* (ICD-9-CM: 483.1) and *Strep*. *pneumoniae* (ICD-9-CM: 041.00–041.09 and 482.3) have been recorded inadequately in this data set. Thus, additional clinical studies are required for verifying the association between the etiology of pneumonia pathogens and liver abscess.

Another explanation for the development of PLA was the dysfunction of gut barriers during pneumonia. A previous study demonstrated that enterocyte injury is common in patients critically ill with pneumonia [27]. Therefore, after gut barrier dysfunction, bacteria may translocate to the liver through the portal vein system, eventually causing abscess formation in liver. DM patients are more likely to develop PLA because of their higher gut permeability [28]. Moreover, antibiotic therapy during pneumonia, which alters the composition and functions of gut microbiota and the intestinal immune system, eventually increases the risk of bacterial invasion [29]. Another potential mechanism contributing to PLA is the direct influence of pneumonia on the immune system. An immunocompromised status is a well-known potential risk factor for PLA. Studies have demonstrated that apoptosis of neutrophils and necroptosis of macrophages are common during bacterial pneumonia [30, 31].

A critical finding reported in this study is that hospitalization for pneumonia within 180 days before the index date was associated with an increased PLA risk. Our results also indicated that increased hospitalization for pneumonia was correlated with higher PLA risk. These findings are of clinical significance to physicians because a timely diagnosis of PLA is difficult [32]. The most common signs and symptoms of PLA, such as fever and abdominal pain, are nonspecific; even laboratory findings are nonspecific and non-diagnostic. PLA is usually diagnosed using imaging techniques such as sonography and computed tomography. This study established an association of pneumonia and development of PLA. A follow up imaging in selected patients treated for pneumonia may lead to early diagnosis of PLA especially if they have history of gall stones and symptoms suggestive of PLA.

The strength of this case–control study is that we used data from LHID2010, the subset of Taiwan National Health Insurance Research Database, a nationwide dataset comprising 1 million beneficiaries randomly selected from the 2010 NHI registry. The Taiwan NHI system, established in 1995, covers the medical expenses of approximately 98% of the population of Taiwan; therefore, the included data accurately represents conditions in Taiwan. Moreover, the LHID2010 has a longitudinal design to minimize selection biases. Furthermore, the possibility of recall bias was reduced by using the administrative database for analysis. In fact, several high quality articles publications were generated by this database set [33–35].

Our study also has several limitations. First, the LHID2010 does not contain information regarding patients’ clinical presentation, laboratory data, microbiological culture data, and pneumonia severity index score; however, the risk of bacteremia is correlated with the severity of pneumonia. Second, although the cases and controls were matched with comorbidities, our sample size was insufficient to match for all comorbidities (Table 1); therefore, we performed multivariate analysis (Table 2, Table 3) to adjust for these potential confounding factors. Third, liver abscess is a disease endemic to Taiwan and East Asian countries; thus, our results may not be generalizable to Western populations. Fourth, small size of patient population and weakness of propensity matching methodology nature which does not account for unmeasured confounders in this study should be taken into consideration.

In conclusion, pneumonia is an independent risk factor for subsequent PLA. Moreover, hospitalization for pneumonia within 180 days before PLA diagnosis was associated with an increased PLA risk. Therefore, clinical physicians should consider PLA as a differential diagnosis for patients with infections after hospitalization for pneumonia.
